# Hypertension evaluated in the public and private Brazilian health system hypertension in public and private service

**DOI:** 10.3389/fcvm.2023.1254933

**Published:** 2023-09-18

**Authors:** Kecia C. F. O. Amorim, Priscila Valverde O. Vitorino, Audes D. M. Feitosa, Mayara Cedrim Santos, Rodrigo Bezerra, Lais Rocha Lopes, Miguel Camafort, Antonio Coca, Ana Luíza Lima Sousa, Weimar K. S. Barroso

**Affiliations:** ^1^ Pós Graduação em Ciências da Saúde, Faculdade de Medicina, Universidade Federal de Goiás, Goiânia, Brasil; ^2^ Programa de Pós-graduação Stricto Sensu em Atenção à Saúde, Escola de Ciências Sociais e da Saúde, Pontifícia Universidade Católica de Goiás, Goiânia, Brasil; ^3^ Serviço de Hipertensão de Pernambuco, Procape/UPE, Universidade de Pernambuco, Recife, Brasil; ^4^ Departamento de Clínica Médica, Hospital das Clínicas, EBESERH, Goiânia, Brasil; ^5^ Hypertension Unit, Hospital Clínic, Barcelona University, Barcelona, Spain; ^6^ Liga de Hipertensão Arterial, Universidade Federal de Goiás, Goiânia, Brasil

**Keywords:** hypertension, public health system, private health system, targets, treatment

## Abstract

**Introduction:**

Hypertension (HT) remains the leading cause of death worldwide. In Brazil it is estimated that 35% of the adult population has HT and that about 20% of these have blood pressure values within the targets recommended for the reduction of cardiovascular risk. There are some data that point to different control rates in patients treated by cardiologists in public and private referral center and this is an important point to be investigated and discussed.

**Objective:**

To compare sociodemographic characteristics, body mass index (BMI), antihypertensive (AH) drugs, blood pressure (BP) and control rate in public (PURC) and private (PRRC) referral centers.

**Methodology:**

A cross-sectional multicenter study that analyzed data from hypertensive patients assisted by the PURC (one in Midwest Region and other in Northeast region) and PRRC (same distribution). Variables analyzed: sex, age, BMI, classes, number of AH used and mean values of systolic and diastolic BP by office measurement and home blood pressure measurement (HBPM). Uncontrolled hypertension (HT) phenotypes and BP control rates were assessed. Descriptive statistics and *χ*^2^ tests or unpaired *t*-tests were performed. A significance level of *p* < 0.05 was considered.

**Results:**

A predominantly female (58.9%) sample of 2.956 patients and a higher prevalence of obesity in PURC (*p* < 0.001) and overweight in PRRC (*p* < 0.001). The mean AH used was 2.9 ± 1.5 for PURC and 1.4 ± 0.7 for PRRC (*p* < 0.001). Mean systolic and diastolic BP values were higher in PURC as were rates of uncontrolled HT of 67.8% and 47.6% (*p* < 0.001) by office measurement and 60.4% and 35.3% (*p* < 0.001) by HBPM in PURC and PRRC, respectively.

**Conclusion:**

Patients with HT had a higher prevalence of obesity in the PURC and used almost twice as many AH drugs. BP control rates are worse in the PURC, on average 15.3 mmHg and 12.1 mmHg higher than in the PRRC by office measurement.

## Introduction

Hypertension (HT) remains the leading cause of death worldwide. Despite this, it remains underdiagnosed and undertreated (1). In Brazil, it is estimated that 35% of the adult population has HT and that about 20% of these have blood pressure (BP) values within the targets recommended for the reduction of cardiovascular (CV) risk (2).

However, when we evaluate the rates of HT control in patients treated by cardiologists and in private services, the values found are better than those described in the databases of patients assisted in the Unified Health System (SUS) and can reach 60.6% (3–6).

Further, it is well established that both diagnosis and assessment of HT control by BP monitoring methods are more accurate than office measurement and should be performed whenever possible (2, 7, 8).

Given this scenario this study aimed to compare BP values and BP control among hypertensive patients followed up in public referral center (PURC) and private referral centers (PRRC), located in two regions of Brazil (Midwest and Northest) under the coordination of the same medical team and following the same treatment protocols established by the Brazilian Guidelines on Hypertension (2).

## Methods

### Study design and participants

A cross-sectional multicenter study that analyzed data from patients with HT assisted by the PURC and PRRC obtained from an online platform (*telemrpa.com.br*) between the years 2017 and 2021. The BP values were recorded and stored in the equipment memory and were then included in the TeleMRPA® platform, a telemedicine tool for providing remote reports.

Participants needed to be 18 years of age with a diagnosis of HT and in the use of antihypertensive (AH) drugs to be included. All patients with these criteria were included. The sample was calculated considering a prevalence of HT in Brazil of 32.3% (2). A minimum sample of 583 participants was obtained. This study was submitted to and approved by the Ethics Committee on Human Research of the Hospital das Clínicas of the Universidade Federal de Goiás (registration number: CAAE 99691018.7.0000.5078), which waived the need for an informed consent form.

### Variables

Baseline clinical variables were collected at the time of home blood pressure measurement (HBPM) and comprised the following data: age, sex, body mass index (BMI) using Quetelet's formula (9), classes and number of AH used, mean values of systolic blood pressure (SBP) and diastolic blood pressure (DBP) by office measurement and by HBPM.

Nutritional status was classified as overweight (yes or no), obesity (yes or no). and overweight (yes or no). Overweight was considered as those classified as overweight and obese I, II or III (9).

OMRON brand automatic digital devices were used for BP measurements. The HT was standardized through the protocols of the Brazilian Guidelines for Ambulatory Blood Pressure Monitoring and Guidelines for Residential Monitoring of Blood Pressure 2018 (7). Six measurements were performed per day, three in both the morning (upon waking) and evening/night (before dinner or two hours after), respectively, with one-minute intervals for a total of 24 valid measurements being standardized to at least 14 as an acceptable quality standard. Discrepant measurements such as SBP > 250 mmHg or <70 mmHg; DBP > 140 mmHg or <40 mmHg and pulse pressure (PP) >100 mmHg or <20 mmHg were excluded. Controlled HT was defined as SBP < 140 mmHg and DBP < 90 mmHg considering office measurement or SBP < 130 mmHg and DBP < 80 mmHg by HBPM (2).

### Statistical analysis

Continuous variables with normal distribution were presented as mean and standard deviation while those without normal distribution were presented as median (25th, 75th percentiles). Categorical variables were presented as proportions.

To compare the variables between the groups studied the following statistical tests were used: *Student'st*-test for continuous variables with normal distribution, *Mann-Whitney test* for continuous variables without normal distribution and *χ*^2^ for categorical variables. A-value of *p* < 0.05 was considered statistically significant. Analyses were performed using STATA version 14 *software*.

## Results

A total of 2.956 participants were evaluated, of which 1.789 (60.5%) and 1.167 (39.5%) were from the PRRC and PURC groups respectively with no age difference. The frequency of males was higher in the private service (Table 1).

**Table 1:** Comparison of sociodemographic characteristics between private and public referral centers.

	Total (*n* = 2,956)	Private (*n* = 1,789)	Public (*n* = 1,167)	
Sex (*n* = 2,955)				<0.001
Female	1.742 (58.9)	975 (54.5)	767 (65.7)	
Male	1.213 (41.1)	813 (45.5)	400 (34.3)	
Age (years)	58.8 ± 12.2	58.6 ± 12. 8	59.3 ± 11.3	0.183
Age Group				0.080
18–59 years old	1.419 (48.0)	882 (49.3)	537 (46.0)	
60 years or older	1.537 (52.0)	907 (50.7)	630 (54.0)	
Obesity				<0.001
No	1.826 (62.6)	1.172 (65.5)	654 (58.0)	
Yes	1.090 (37.4)	617 (34.4)	473 (42.0)	
Overweight				<0.208
No	633 (21.7)	402 (22.5)	231 (20.5)	
Yes	2.283 (78.3)	1.837 (77.5)	896 (79.5)	
Nutritional status				<0.001
Normal weight	633 (21.7)	492 (22.4)	231 (20.5)	
Overweight	1.193 (40.9)	770 (43.0)	423 (37.5)	
Obesity	1.090 (37.4)	617 (34.5)	473 (42.0)	
Number of medications				
BRA	1.762 (59.6)	1.063 (59.4)	699 (59.9)	0.796
IECA	840 (28.4)	502 (28.0)	338 (28.9)	0.595
Thiazide Diuretic	1.048 (35.4)	398 (22.5)	650 (55.7)	<0.001
BCC	1.212 (41.0)	654 (36.7)	558 (47.8)	<0.001
Beta-blocker	960 (32.5)	381 (21.3)	579 (49.6)	<0.001
Potassium saver	268 (9.1)	60 (3.3)	208 (17.8)	<0.001
Alpha 2 agonists	206 (7.0)	31 (1.7)	175 (15.0)	<0.001
Vasodilators	93 (3.1)	4 (0.2)	89 (7.6)	<0.001
Loop Diuretic	107 (3.6)	9 (0.5)	98 (8.4)	<0.001

*Χ*^2^, ARB, angiotensin receptor blocker; ACEI, angiotensin-converting enzyme inhibitor; CCB, calcium channel blocker.

As for nutritional status, only 21.7% of the sample was not overweight with a higher prevalence of obesity in the PURC compared to the PRRC.

Regarding antihypertensive drugs, the use of angiotensin-converting enzyme inhibitors (ACEI) and angiotensin receptor blockers (ARB) was similar in both groups. However, all other classes have a higher frequency of use in patients followed up in the public service. The mean number of antihypertensives used was 2.0 ± 1.3 for the total sample; 1.4 ± 0.7 for PRRC and 2.9 ± 1.5 for PURC (p < 0.001) (Table 1).

**Figure 1:**
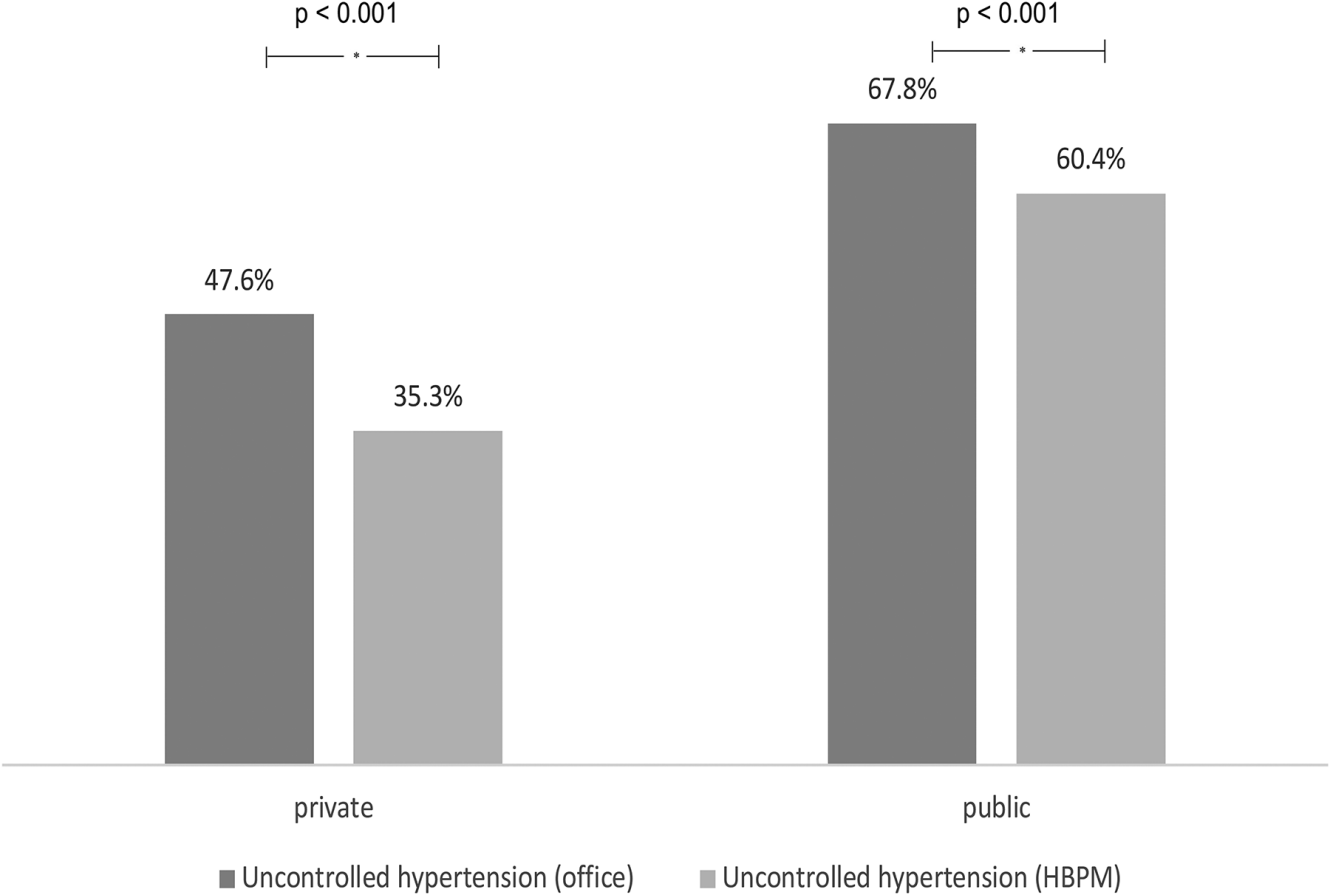
Comparison of the frequency of uncontrolled hypertension between public and private services considering office measures and home blood pressure measurement (HBPM).

All mean blood pressure values, both from office measurements and HBPM in the PURC and PRRC are described in Table 2. The frequency of uncontrolled HT was higher in the PURC both by office measurements and by HBPM (Figure 1).

**Table 2:** Comparison of body mass index and pressure values between private and public referral centers.

	Total	Private	Public	*p*
BMI (Kg/m^2^)	28.9 ± 5.2	28.6 ± 4.8	29.4 ± 5.7	**<0**.**001**
Office pressure				
SBP (mmHg)	135.9 ± 21.9	130.6 ± 17.2	144.0 ± 25.5	**<0**.**001**
DBP (mmHg)	84.0 ± 12.3	81.9 ± 10.2	87.3 ± 14.4	**<0**.**001**
HBPM				
SBP (mmHg)	127.7 ± 17.6	123.6 ± 13.6	134.0 ± 20.8	**<0**.**001**
DBP (mmHg)	78.9 ± 11.0	76.9 ± 8.9	82.0 ± 13.0	**<0**.**001**
PP (mmHg)	53.3 ± 14.4	51.0 ± 12.6	56.6 ± 16.2	**<0**.**001**
Max morning SBP (mmHg)	128.2 ± 18.0	123.9 ± 14.1	134.7 ± 21.2	**<0**.**001**
Max morning DBP (mmHg)	80.0 ± 11.4	77.9 ± 9.5	83.1 ± 13.3	**<0**.**001**
SBP variability morning	8.18 ± 3.6	7.5 ± 3.2	9.2 ± 3.9	**<0**.**001**
PAD variability morning	4.8 ± 2.5	4.6 ± 2.3	5.4 ± 2.6	**<0**.**001**

Unpaired *t*-test. BMI, body mass index; HBPM, residential blood pressure monitoring; SBP, systolic blood pressure; DBP, diastolic blood pressure; PP, pulse pressure.

Bold values are statistically significant.

**Figure 2:**
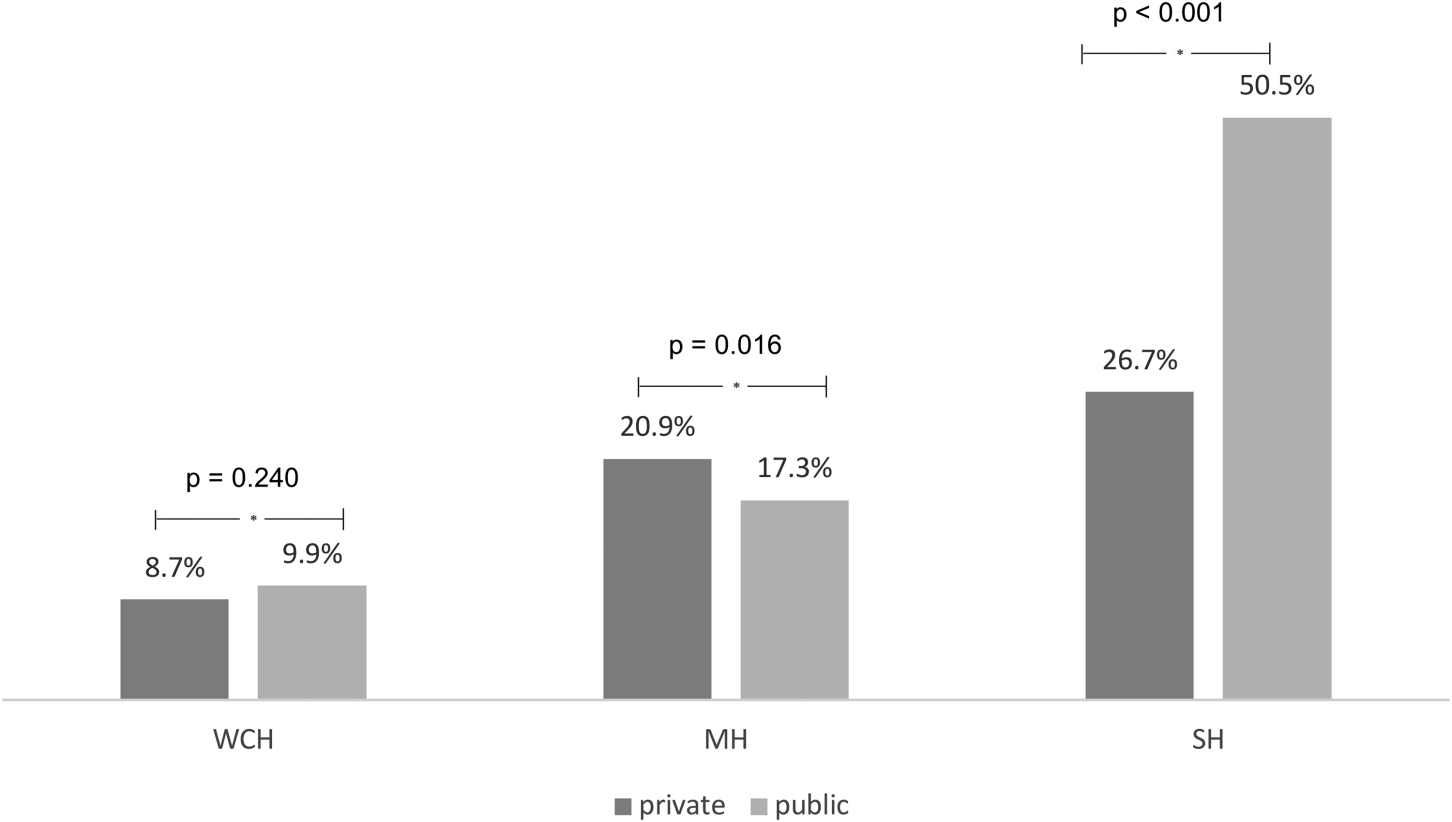
Comparison of the frequency of hypertension phenotypes between public and private services considering office measures and that of residential blood pressure monitoring. WCH, whit-coat hypertension; MH, masked hypertension; SH, sustained hypertension.

Regarding the possible phenotypes of uncontrolled BP in treated patients with HT, the frequency of sustained hypertension (SH), when both office BP and HBPM are above the recommended targets, was higher in the SRPU. However, masked hypertension (MH) was more frequent in PRRC (Figure 2).

**Figure 3:**
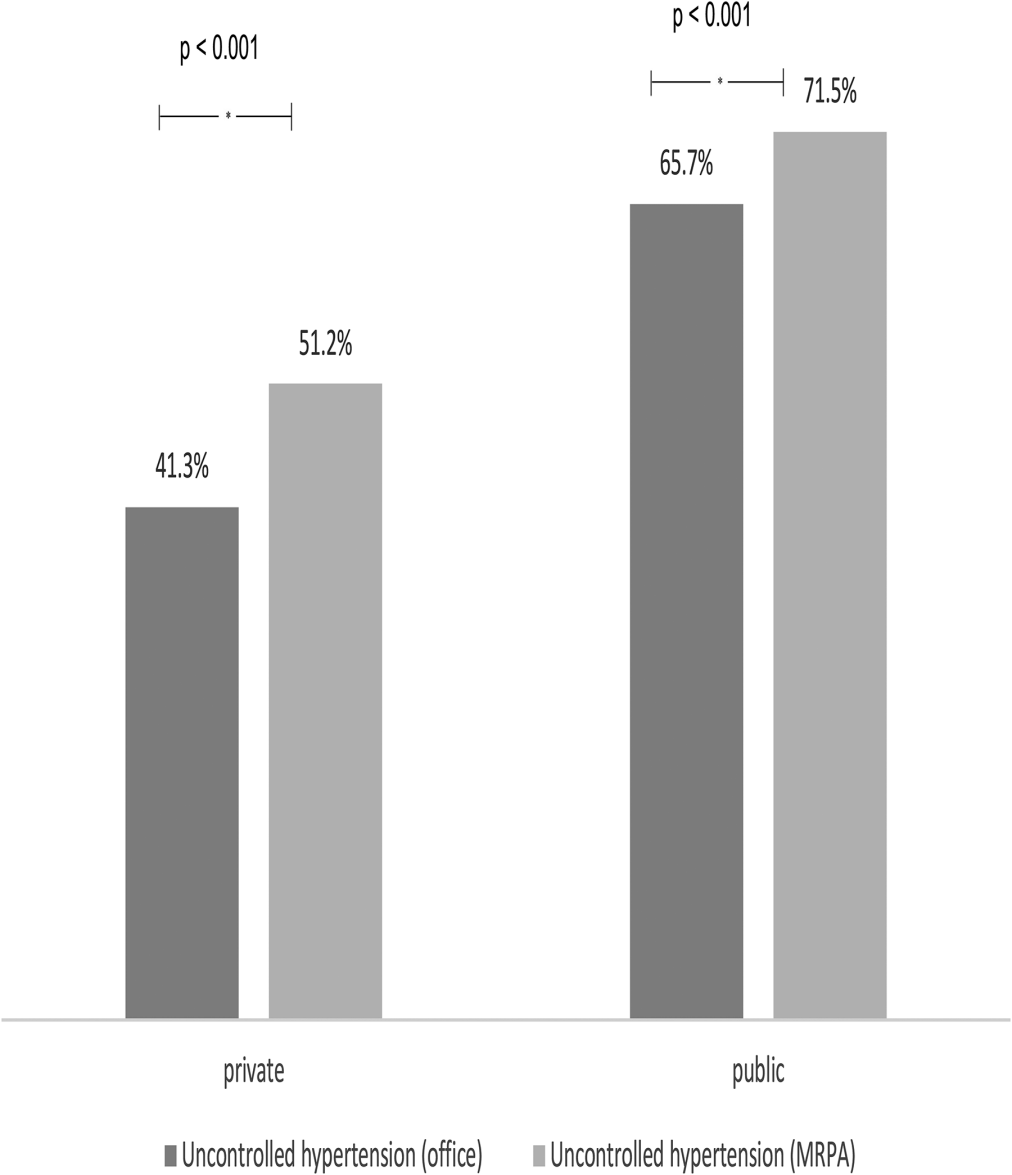
Comparison of the frequency of uncontrolled hypertension in patients using more than three medications between public and private services considering office measurements and HBPM.

Furthermore, we evaluated all 904 patients (30.6%) who were using 3 or more classes of antihypertensive drugs in combination. Further, we found this subgroup had a higher prevalence of uncontrolled HT in the PURC (Figure 3).

## Discussion

The sample evaluated consisted of people with HT with an average age of just under 60 years and an overweight average body mass index. We observed a higher prevalence of females in the total sample, with an even higher prevalence in the PURC. This characteristic of higher frequency of medical care by females has already been described in other publications and denotes the lack of attention to men's health concerning HT (10, 11).

Another important aspect observed in the sample was the higher overweight prevalence in the PRRC (43.0%) and obesity in the PURC (42%). Such characteristics may denote a poorer quality diet and an increased association with comorbidities or severity of hypertensive disease in patients in the public service because it is known that increased BMI and abdominal circumference are risk factors for both increased BP and major cardiovascular diseases (12–14). Moreover, socioeconomic and cultural barriers, in general, are associated with the differences in BMI found both in our sample and in other analyses already published (15, 16).

PURC patients used a greater number of antihypertensive medications. Moreover, except for the classes that block the renin-angiotensin-aldosterone system (ACEI or ARB), the other classes were used more frequently in the PURC. As for antihypertensives available for use in the basic health network in our country, most of them have short half-lives and we do not have fixed combinations in a single pill. This reality leads to the need for the use of a greater number of drugs, pills and daily doses. This is known to be associated with lower adherence to treatment and resulting worsening in BP control (2, 5, 17, 18). We should also consider that there was a higher prevalence of obesity in the PURC, a factor associated with pressure levels that are more difficult to control (2, 5, 17, 18).

Despite the greater number of AH used in PURC, the mean SBP and DBP values, whether measured in the office or by HBPM remained significantly higher than in PRRC. The consequence of a higher number of uncontrolled patients is an increase in the incidence of major associated CV outcomes. Effectively improving the control of HT means dramatically reducing the incidence of stroke, acute myocardial infarction, heart failure and chronic kidney disease (19–21).

When we evaluated the possible phenotypes of uncontrolled HT, we found a higher prevalence of SH in the PURC. This phenotype is the one with the worst prognosis relating to cardiovascular outcomes in hypertensive patients since it denotes a lack of BP control in both office measurements and HBPM (22). This aspect reinforces the importance of assessment, whenever possible, through BP monitoring methods. This way, we will have a broader understanding of the BP control status and the best strategies for pharmacological treatment (23). In a previous study of patients with HT treated by specialist physicians and evaluated by HBPM, SH rates were 33.7% (6); in our sample we found values of 50.7% and 26.7% in PURC and PRRC, respectively (*p* < 0.001).

When evaluating the subgroup using three or more AH drugs, we found higher levels of patients off target by both the office measure and HBPM which was to be expected and similar to the total sample, those followed up in the PURC had worse control rates than the PRRC group.

This study has some limitations since we have not evaluated socio economic level and adhesion with specific tools, however, by evaluating a population of more than 3,000 hypertensive patients using public and private reference centers under the same coordination, we believe it is possible to evaluate with less bias the aspects related to drug use in both scenarios and the impact on BP control. Furthermore, the information obtained both by the office measurement and by the HBPM allows greater accuracy in the evaluation of the recommended goals.

It was possible to observe that patients followed up in public referral centers have a higher prevalence of obesity and use more than double the number of antihypertensive drugs than private services.

The blood pressure control rates assessed by both office measurement and HBPM are always worse in the PURC and on average 15.3 mmHg and 12.1 mmHg higher than in the PRRC for SBP and DBP, respectively, in the office measurements.

The poorer control of HT is associated with a higher incidence of the main cardiovascular outcomes. Therefore, there is an urgent need to reassess strategies so that, in the end, we can decrease the disparities between public and private services and increase our hypertensive population's cardiovascular protection.

## Data Availability

The raw data supporting the conclusions of this article will be made available by the authors, without undue reservation.
